# The Mother-Newborn Skin-to-Skin Contact Questionnaire (MSSCQ): development and psychometric evaluation among Iranian midwives

**DOI:** 10.1186/1471-2393-14-85

**Published:** 2014-02-24

**Authors:** Fatemeh Nahidi, Sedigheh Sadat Tavafian, Mohammad Heidarzadeh, Ebrahim Hajizadeh, Ali Montazeri

**Affiliations:** 1 Department of Health Education, Faculty of Medical Sciences, Tarbiat Modares University, Tehran, Iran; 2 Department of Neonatal Health, Ministry of Health & Medical Education, Tehran, Iran; 3 Maternal, Fetal and Neonatal Research Center (MFNRC), Tehran University of Medical Sciences (T.U.M.S), Tehran, Iran; 4 Department of Biostatistics, Faculty of Medical Sciences, Tarbiat Modares University, Tehran, Iran; 5 Mental Health Research Group, Health Metrics Research Center, Iranian Institute for Health Sciences Research, ACECR, Tehran, Iran

## Abstract

**Background:**

Despite the benefits of mother-newborn skin-to-skin contact immediately after birth, it has not been universally implemented as routine care for healthy term neonates. Midwifes are the first person to contact the neonate after birth. However, there is evidence that many midwives do not perform mother-newborn skin-to-skin contact. The aim of this study was to develop and psychometrically evaluate an instrument for measuring factors associated with mother-newborn skin-to-skin contact (MSSCQ) based on the PRECEDE-PROCEED model.

**Methods:**

This was a two-phase qualitative and quantitative study. It was conducted during 2010 to 2012 in Tehran, Iran. In the qualitative part, 150 midwives working in labor room participated in 19 focus group discussions in order to generate a preliminary item pool. Then, content and face validity were performed to provide a pre-final version of the questionnaire. In the quantitative phase, reliability (internal consistency and test-retest analysis), validity and factor analysis (both exploratory and confirmatory) were performed to assess psychometric properties of the instrument.

**Results:**

A 120-item questionnaire was developed through the qualitative phase. It was reduced to an 83-item after content validity. The exploratory factor analysis loaded fifteen-factors and three constructs (predisposing, enabling and reinforcing) containing 82 items (38, 18, and 26 statements, respectively) that jointly accounted for 60.61% of observed variance. The Confirmatory factors analysis determined a model with appropriate fitness for the data. The Cronbach’s alpha coefficient showed excellent internal consistency (alpha = 0.92), and test-retest of the scale with 2-week intervals indicated an appropriate stability for the MSSCQ (ICC = 0.94).

**Conclusion:**

The Mother-Newborn Skin-to-Skin Contact Questionnaire (MSSCQ) is a reliable and valid theory-based measurement and now can be used in clinical practice, midwifery and nursing studies.

## Background

Early neonatal care is of utmost importance [1]. One example of such care is the mother-newborn skin-to-skin contact immediately after birth in healthy term neonates [2]. Findings of studies over the last 25 years suggest that the first hour after birth is a critical time for bonding between mother and child, when both are ready for a coordinated reciprocal interaction [3-8]. Instinctive nourishing behaviors, including seeking and breastfeeding, start in this time [9]. Another advantage is the improvement in mother’s ability for caring for her child [10], attachment between mother and newborn and the long term positive impact of attachment behaviors [11-13], reduced stress of mother and newborn, finding ways to counter stress [11], regulation of breathing, heartbeat, and body temperature of the newborn, calm sleep, shortened interval between delivery and breastfeeding, success in first breastfeeding, elongation of breastfeeding period [14], regulation of neonatal blood sugar level and reduced child cries [15], earlier discharge of mother and newborn, and reduced behavioral problems [13]. Despite the large quantity of evidence suggesting the positive impact of immediate mother and neonate skin contact, it has not been adopted as a universal post-delivery care for healthy term children [16]. Skin contact is a simple and cost-effective method for improving post-delivery care, encouraging exclusive breastfeeding, and increasing the duration of breastfeeding by midwives [13].

Midwifery is an important occupation for labor and social health, and providing obstetrical counseling services. According to an unofficial report by the Iranian Ministry of Health, for the year 1999 to 2009 there were 32,228 midwifery graduates in different educational levels. The same source estimated that the number of midwifery graduates increased to 55 thousand by 2012. There is evidence that in Iran 54.45% of deliveries (48.60% in cities and 64.32% in villages) is performed by midwifes [17] and they are the first person to contact the neonate after birth. However, by our own experiences we observed that about 90% of midwives do not perform mother-newborn skin-to-skin contact while the Iranian Ministry of Health asks that all midwives should perform skin-to-skin contact immediately after birth. Therefore, we thought it is necessary to identify factors that prevent midwives to perform skin-to-skin contact. To elucidate such factors we decided to use a theoretical framework that might help to formulate the issue. As such the Precede-Proceed model was selected. Green and Kreuter developed this model in 1970 and states that in order to modify a behavior, the individual alone should not be targeted; rather, the entire surrounding environment and the factors affecting his/her behavior should be considered [18-20]. The model consists of several parts including a construct namely educational and ecological assessment. The educational and ecological assessment by itself consists of three factors: predisposing factors, enabling factors and reinforcing factors. To the best of our knowledge no study or instrument was developed to deal with the factors associated with mother-newborn skin-to-skin contact immediately after birth for midwives in Iran or elsewhere. Thus the main objective of this study was to develop an instrument for measuring the above-mentioned factors that are associated with mother-newborn skin-to-skin contact.

## Methods

### Design

This was a two-phase study. First we conducted a qualitative study to generate an item pool. Then, a quantitative approach was used to evaluate the questionnaire.

### Item generation

In all 19 Focus Group Discussions (FGD) with 150 midwives working in labor rooms were held to elucidate what issues are important in mother-newborn skin-to-skin contacts in order to generate an item pool for developing a questionnaire on the topic. We have tried to recruit midwives with different characteristics to ensure that diverse demographic backgrounds are present in the focus groups. The number of participants in each session was 6-12 individuals and each session lasted for 1.5-2 hours. All midwives informed about the aim of the study. After participants’ consent, all discussions were tape-recorded. The discussions were held in the hospitals. Participants were asked whether they are familiar with the issue of mother-newborn skin-to-skin contact. In addition they were asked about possible benefits of skin-to-skin contacts for mother and newborns. Factors associated with skin-to skin contacts and potential barriers for performing skin-to-skin contact also were discussed. Furthermore the main investigator (FN) observed and recorded all behaviors and nonverbal messages of the participants closely. We used a help sheet for discussion sessions. We stopped data collection until saturation was reached. Subsequently, all sessions were transcribed and were checked twice for accuracy. The conventional content analysis [21] was performed to elucidate the semantic units. Consequently the condensed semantic units were provided and each one represented as an item for inclusion in the study questionnaire. For instance a midwife stated that ‘Skin-to-skin contact has several benefits for newborn’s health’. As a result the condense unit of meaning as an item in the questionnaire was: ‘Skin contact improves neonate’s physical health’. At last, the data derived from the qualitative phase were crosschecked and in all 120 items were generated. Finally content and face validity were performed in order to provide the pre-final version of the questionnaire.

#### Content validity

In this stage, to determine the content validity we used both qualitative and quantitative methods. For qualitative method an expert panel consisting of 15 specialists, including 5 neonatologists, 3 obstetricians, 1 epidemiologist, 3 midwifery teachers, 1 health education expert, and 2 experts in qualitative methods evaluated the questionnaire for ‘grammar’, ‘wording’, ‘item allocation’, and ‘scaling’ indices [22,23]. The expert panel checked all items and inserted their recommendations into the questionnaire. For calculating the quantitative content validity, we used the Content Validity Ratio (CVR) and the Content Validity Index (CVI). The necessity of an item was assessed using a 3-point rating scale: a) *essential,* b) *useful but not essential,* c) *unessential* in order to calculate the CVR [22,23]. Then, based on the Lawshe’s table, items with CVR value of 0.4 or above were considered acceptable [24,25]. For the CVI, according to Waltz & Bausell’s recommendation, the same experts were asked to evaluate the items based on a 4-point Likert scale on, *a)* simplicity, *b)* relevancy, and *c)* clarity [26,27]. The CVI value of 0.79 or above was considered satisfactory for each statement [22,23,28].

#### Face validity

We applied both quantitative and qualitative methods for performing face validity. For the purpose of qualitative approach, we asked 7 midwives to assess each item for “ambiguity”, “relevancy”, and “difficulty”. For quantitative approach, the same midwives were asked to evaluate the questionnaire and score the importance of each item on a 5-point Liker scale in order to calculate the impact score for each item. It was calculated as multiplying the importance of an item with its frequency [Impact Score = Frequency (%) × Importance). The impact score of 1.5 or above was considered satisfactory as recommended [22,29]. In conclusion 36 items were removed and the pre-final version of the questionnaire consisting of 84 items was provided for the main study [Additional file 1].

### The main study and data collection

A multi stage cluster sampling was applied. First Tehran (the capital of Iran) was divided into 5 regions: north, south, west, east and center and all hospitals located in these 5 regions were identified. Then from each region, three hospitals were randomly selected. The sample size was estimated on the basis of our planned procedure for exploratory factor analysis. Assigning 3 individuals to each item, a sample size of 252 was estimated (84 × 3) [22]. Considering the possible attrition, we planned to recruit a sample of 300 midwives from 15 hospitals working in labor or operating rooms of hospitals. In addition to the study questionnaire the demographic characteristics of midwives including age, work experience, employment status, marital status, academic degree in midwifery, parity and midwives’ interests in work environment were also collected.

### Statistical analysis

Several statistical analyses were performed to assess the psychometric properties of the questionnaire. These are explained as follows:

1. Construct validity: The construct validity of the questionnaire was performed using both exploratory (EFA) and confirmatory factor analyses (CFA) [22,24,26].

2. Reliability: Internal consistency of the instrument was evaluated by the Cronbach’s alpha coefficient, once for the entire questionnaire, once for each construct, and once for each factor. The Cronbach’s alpha coefficient of 0.7 or above was though satisfactory [22,36]. In addition, we used test-retest to examine the instrument’s stability by calculating Intraclass Correlation Coefficient (ICC) with a sub-sample of midwives (n = 30) that completed the questionnaire twice with an interval of 2-weeks [37-40]. The acceptable value for ICC, was considered 0.4 or above [39]. All the statistical analyses and confirmatory factor analyses were performed using the SPSS version 18.0 [41] and the LISREL 8.80 for Windows, respectively [41,42].

**Figure 1:**
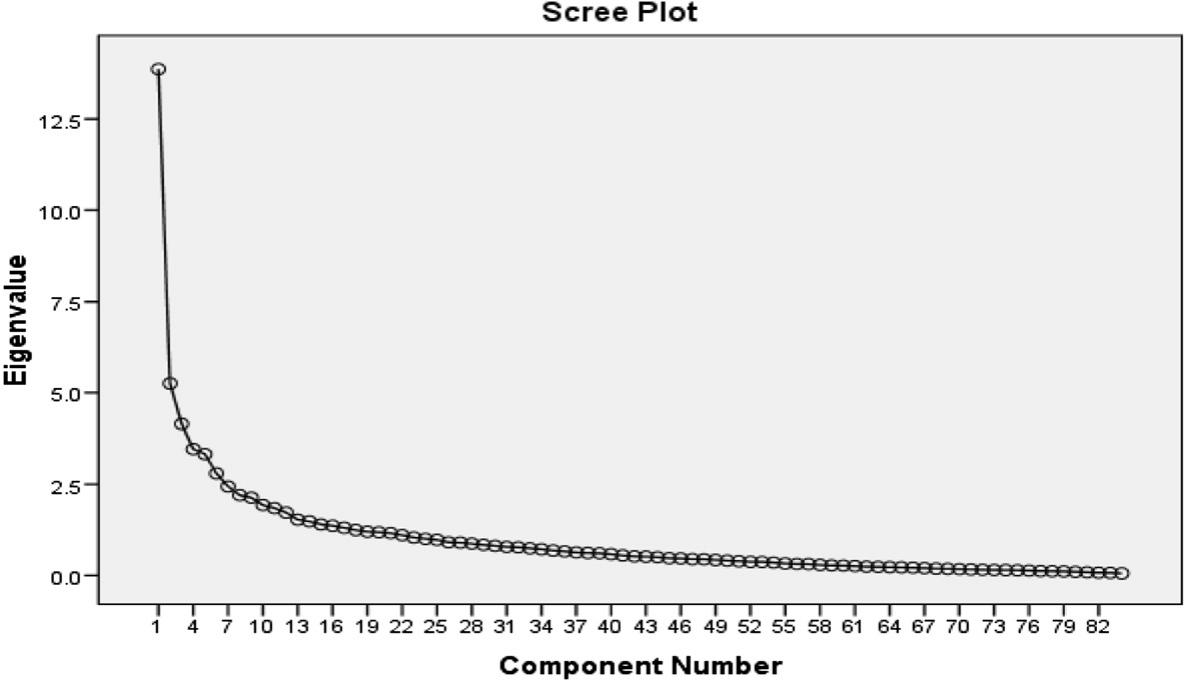
Scree plot for determining factors of the designed instrument.

*Exploratory factor analysis:* the principal component analysis with varimax rotation was performed to extract underlying factors. Factor loadings equal or greater than 0.3 were considered appropriate and eigenvalues above 1 and scree plot were used for determining the number of factors (Figure 1) [30]. The Kaiser-Meyer-Olkin (KMO) and Bartlett’s Test of Sphericity were used to assess the appropriateness of the sample for the factor analysis [30,31].

*Confirmative factor analysis:* confirmative factor analysis was performed for comparing and assessing the model fitness [22,31]. As recommended various fit indices including: relative Chi-square, Root Mean Square Error of Approximation (RMSEA), Goodness of Fit Index (GFI), Comparative Fit Index (CFI), Non-Normed Fit Index (NNFI), Normed Fit Index (NFI), Standardized Root Mean Square Residual (SRMR) were used [31,32]. The cut-off values for GFI, CFI, NNFI and NFI could range between ‘0-1’ [33] but value of 0.90 or more is generally considered to indicate acceptable model fit [31,34,35]. For SRMR, the values below 0.05 indicate good fit but values less than 0.08 and 0.01 indicate adequate fit and are acceptable, respectively [35].

### Ethics

The ethics committee of Trabiat Modares University approved the study. All participants gave informed written consent.

## Results

### Participants

In all, 450 midwives participated in the study (150 midwives in the qualitative study, and 300 midwives in the main study). However, of those who participated in the main study 8 midwives were excluded due to incomplete questionnaires and the data obtained from the remaining 292 midwives were analyzed. The mean age of midwives was 36.06 (SD = 8.4) years, and their mean work experience was 11.07 (SD = 8.29) years. The demographic and obstetric characteristics of midwives are shown in the Table 1.

**Table 1:** The characteristics of the study sample

	**Qualitative sample (n = 150)**	**EFA and CFA sample (n = 292)**	**Test-retest sample (n = 30)**	
Age (years)				
Mean (SD)	38.27(7.9)	36.06(8.72)	36.20(7.20)	
Work experience (years)				
Mean (SD)	13(8.1)	11.07(8.29)	10.67(7.37)	
Employment status	Number (%)	Number (%)	Number (%)	
Official	84(56)	124(42.5)	16(53.3)	
Contractual	7(4.7)	29(9.9)	10(33.3)	
By project	41(27.3)	92(31.5)	2(6.7)	
Mandatory service	18(12)	47(16.1)	2(6.7)	
Marital status	Number (%)	Number (%)	Number (%)	
Single	30(20)	111(38.1)	8(26.7)	
Married	117(78)	180(61.6)	21(70.0)	
Divorced/Widow	3(2)	1(0.3)	1(3.3)	
Degree in midwifery	Number (%)	Number (%)	Number (%)	
Associate’s Degree	11(7.3)	14(4.8)	2(6.7)	
Bachelor’ Degree	129(86)	257(88.0)	22(73.3)	
Master’ Degree	10(6,7)	21(7.2)	6(20.0)	
Midwives’ interest in work environment	Number (%)	Number (%)	Number (%)	
Yes	118(85.1)	231(79.1)	23(76.7)	
No	32(21.3)	61(20.9)	7(23.3)	
Parity	Number (%) (n = 101)	Number (%) (n = 148)	Number (%) (n = 20)	
Once	39(38.6)	65.(43.9)	8(40.0)	
Twice	53(52.5)	76(51.4)	9(45.0)	
Three and more	9(8.9)	7(4.7)	3(15.0)	

### Exploratory factor analysis

The Kaisar-Meyer-Olkin was 0.763, which falls in the “very good” category and the Bartlett’s test of Sphericity was significant (chi-square = 14052.735, p < 0.0001) indicating adequacy of samples for Explorative Factor Analysis. The initial analysis indicated a 16-factors structure for the questionnaire. However, as only one statement was in factor 16, and it overlapped with another factor, the factor was removed. In addition 1 item was not loaded on any factors and thus it was removed. A final 82-item questionnaire loaded on fifteen factors and three distinct constructs as follows:

Predisposing construct: 6-factors, 38-statements,

Enabling constructs: 3-factors, 18-statements and

Reinforcing construct: 6-factors, 26-statements that jointly accounted for 60.61% of variance observed (Tables 2, 3 and 4).

**Table 2:** Predisposing factors derived from principle factor analysis with varimax rotation for the MSSCQ

***Factor 1: Midwife’s attitude with 11 statements***	***Loadings***
1. Skin contact improves mother’s physical health.	0.647
2. Skin contact improves neonate’s physical health.	0.572
3. Skin contact makes mother take better care of the child.	0.764
4. Skin contact improves mother’s success in breastfeeding.	0.727
5. Skin contact improves mother’s satisfaction.	0.803
6. Skin contact improves mother’s mental health.	0.835
7. Skin contact establishes verbal/emotional bonding between midwife and mother.	0.592
8. Skin contact creates a sense of security in the newborn.	0.703
9. Skin contact enhances mother’s love for the newborn.	0.806
10. Skin contact reduces mother’s stress.	0.673
11. Being skilled in performing skin contact by midwife improves the results.	0.479
Test- re-test*	*ICC = 0.842*
*Cronbach’s α coefficient of Factor 1*	*0.92*
*Eigen values*	*13.857*
*Explained variance (%)*	*16.50*
***Factor 3: Newborn’s health with 7 statements***	***Loadings***
12. Skin contact improves newborn’s immunity system.	0.562
13. Skin contact improves the development of the newborn.	0.471
14. Skin contact establishes an emotional bond between parents and the newborn.	0.446
15. Skin contact regulates the newborn’s blood oxygen level.	0.428
16. Skin contact regulates the newborn’s heartbeat.	0.773
17. Skin contact improves the newborn’s breathing.	0.774
18. Skin contact regulates the newborn’s body temperature.	0.695
Test- re-test*	*ICC = 0.996*
*Cronbach’s α coefficient of Factor 3*	*0.81*
*Eigen values*	*3.464*
*Explained Variance (%)*	*4.94*
***Factor 5: Mother’s physical health with 4 statements***	***Loadings***
19. Skin contact accelerates placental delivery.	0.696
20. Skin contact accelerates the uterus’s return to normal.	0.812
21. Skin contact promotes oxytocin release in mother.	0.730
22. Skin contact reduces post-labor bleeding.	0.795
Test- re-test*	*ICC = 0.977*
*Cronbach’s α coefficient of Factor 5*	*0.84*
*Eigen values*	*3.328*
*Explained variance (%)*	*3.96*
***Factor 6: Midwife’s belief about obstacles of performing skin contact with 5 statements***	***Loadings***
23. The newborn’s ill situation hinders skin contact.	0.786
24. Skin contact is not feasible for ill mothers.	0.776
25. Problems of mothers undergoing C-section affect skin contact.	0.823
26. Problems of neonates born to C-section affect skin contact.	0.784
27. Mother’s fatigue caused by nonstandard intervention during labor affects skin contact.	0.652
Test- re-test*	*ICC = 0.989*
*Cronbach’s α coefficient of Factor 6*	*0.84*
*Eigen values*	*2.800*
*Explained variance (%)*	*3.33*
***Factor 7: Midwife’s belief in self-efficacy with 7 statements***	***Loadings***
28. I believe skin contact is essential.	
29. I believe skin contact entails positive results.	0.535
30. I believe skin contact is important.	0.711
31. I believe I can perform skin contact with minimum facilities.	0.345
32. I believe my recommendations for skin contact are acceptable for the mother.	0.402
33. I believe I can use my knowledge to perform skin contact.	0.573
34. I believe in positive results of the skin contact and I perform it.	0.581
Test- re-test*	*ICC = 0.980*
*Cronbach’s α coefficient of Factor 7*	*0.76*
*Eigen values*	*2.441*
*Explained variance (%)*	*2.91*
***Factor 8: Mental health with 4 statements***	***Loadings***
35. Skin contact establishes an emotional bond between mother and newborn.	0.640
36. Skin contact improves the acceptance of motherhood role by the mother.	0.750
37. Skin contact creates a sense of security in mother and newborn.	0.659
38. Skin contact results in future attachment between mother and child.	0.612
Test- re-test*	*ICC = 1.000*
*Cronbach’s α coefficient of Factor 8*	*0.73*
*Eigen values*	*2.203*
*Explained variance (%)*	*2.62*
***Total test-re test of predisposing factors structure***	***ICC = 0.995***
***Total Cronbach’s α coefficient of predisposing factors structure***	***0.89***
***Cumulative Variance (%)***	***44.64***

*Test re-test stability with a 2-week interval (n = 60).

**Table 3:** Enabling factors derived from principle factor analysis with varimax rotation for the MSSCQ

***Factor 13: Managerial-planning with 4 statements***	***Loadings***
1. Presence of a supportive program in the ministry improves skin-to-skin contact.	0.550
2. Skill-teaching programs in hospital improve skin-to-skin contact.	0.609
3. Placing skin-to-skin contact in policies of the ministry of health will improve its implementation.	0.479
4. Encouraging the midwife by hospital authorities will improve skin-to-skin contact.	0.375
Test- re-test*	*ICC = 1.000*
*Cronbach’s α coefficient of Factor 13*	*0.64*
*Eigen values*	*1.531*
*Explained variance (%)*	*1.82*
***Factor 12: Service provided to mother with 5 statement***	***Loadings***
5. Physiologic delivery has a positive impact on skin-to-skin contact.	0.011
6. Encouraging the mother to have skin contact in labor room will improve skin-to-skin contact.	0.209
7. Collaboration of the labor-supporting team improves skin-to-skin contact.	0.680
8. Availability of adequate human resources in labor room improves skin-to-skin contact.	0.653
9. Professional ethical commitment of the midwife improves skin-to-skin contact.	0.446
Test- re-test*	*ICC = 1.000*
*Cronbach’s α coefficient of Factor 12*	*0.50*
*Eigen values*	*1.725*
*Explained variance (%)*	*2.05*
***Factor 2: Preparations with 9 statement***	***Loadings***
10. Educating mothers during pregnancy improves skin-to-skin contact.	0.246
11. Educating companions improves skin-to-skin contact.	0.368
12. Educating the parents before pregnancy improves skin-to-skin contact.	0.339
13. Legalizing skin-to-skin contact improves its implementation in hospitals.	0.639
14. Including skin-to-skin contact in educational curricula of medical and midwifery students will improve its implementation.	0.582
15. Mandating skin-to-skin contact to all hospitals will improve its implementation.	0.699
16. Placing a point for skin-to-skin contact in ranking of hospitals will improve its implementation.	0.821
17. Developing regulations for evaluating midwives based on skin-to-skin contact will improve its implementation.	0.761
18. The supervision of authorities on correct skin-to-skin contact will improve its implementation.	0.709
Test- re-test*	*ICC = 1.000*
*Cronbach’s α coefficient of Factor 2*	*0.85*
*Eigen values*	*5.256*
*Explained variance (%)*	*6.26*
*Cumulative variance (%)*	*22.75*
***Total test-re test of enabling factors structure***	***ICC = 1.000***
***Total Cronbach’s α coefficient of enabling factors structure***	***0.85***

*Test- re-test stability with a 2-week interval (n = 60).

**Table 4:** Reinforcing factors derived from principle factor analysis with varimax rotation for the MSSCQ

***Factor 4: Encouraging factors for midwives with 7 statements***	***Loadings***
1. Encouraging colleagues improves skin-to-skin contact.	0.074
2. Patient’s confidence in the delivery team improves skin-to-skin contact.	0.478
3. Mother’s calmness during skin-to-skin contact will encourage the midwife.	0.654
4. Newborn’s calmness during skin-to-skin contact will encourage the midwife.	0.601
5. Mother’s satisfaction with skin-to-skin contact will encourage the midwife.	0.672
6. Mother’s desire for skin-to-skin contact will encourage the midwife.	0.647
7. Mother’s request for skin-to-skin contact will encourage the midwife to perform it.	0.687
Test- re-test*	*ICC = 1.000*
*Cronbach’s α coefficient of Factor 4*	*0.72*
*Eigen values*	*3.148*
*Explained variance (%)*	*4.12*
***Factor 9: Support of the medical team with 4 statements***	***Loadings***
8. Physician’s support will improve skin-to-skin contact.	0.676
9. Anesthesiologist’s support will improve skin-to-skin contact.	0.753
10. Pediatrician’s support will improve skin-to-skin contact.	0.761
11. Hospital authorities’ support will improve skin-to-skin contact.	0.595
Test- re-test*	*ICC = 1.000*
*Cronbach’s α coefficient of Factor 9*	*0.75*
*Eigen values*	*2.135*
*Explained variance (%)*	*2.54*
***Factor 10: Companion’s support with 3statements***	***Loadings***
12. Presence of educated companion in the labor room improves skin-to-skin contact.	0.439
13. Support of mother’s relatives improves skin-to-skin contact.	0.776
14. The husband’s support improves skin-to-skin contact.	0.776
Test- re-test*	*ICC = 0.889*
*Cronbach’s α coefficient of Factor 10*	*0.68*
*Initial Eigen values*	*1.931*
*Explained variance (%)*	*2.30*
***Factor 11: Self-motivation with 4 statements***	***Loadings***
15. Midwife’s awareness of advantages of skin-to-skin contact improves its implementation.	0.708
16. Midwife’s desire for skin-to-skin contact will encourage her to perform it.	0.683
17. Awareness of advantages of skin-to-skin contact through media will improve its implementation.	0.372
18. Midwife’s support for skin contact will encourage its implementation.	0.526
Test- re-test*	*ICC = 1.000*
*Cronbach’s α coefficient of Factor 11*	*0.66*
*Eigen values*	*1.846*
*Explained variance (%)*	*2.20*
***Factor 14: Facilities and equipment with 5 statements***	***Loadings***
19. Presence of an appropriate labor bed affects skin contact.	0.471
20. The temperature of the labor room affects skin contact.	0.643
21. Availability of private space during labor affects skin contact.	0.557
22. Presence of an appropriate space in the operation room affects skin contact.	0.405
23. Presence of a midwife to take care of the newborn affects skin contact.	0.495
Test- re-test*	*ICC = 1.000*
*Cronbach’s α coefficient of Factor 14*	*0.64*
*Eigen values*	*1.483*
*Explained variance (%)*	*1.77*
***Factor 15: Midwife’s occupational satisfaction with 3 statements***	***Loadings***
24. Midwife’s occupational satisfaction affects skin contact.	0.544
25. Eliminating the marginal responsibilities of midwives affects skin contact.	0.556
26. Providing independence and granting the responsibility of normal delivery to midwife affects skin contact.	0.312
Test- re-test*	*ICC = 0.697*
*Cronbach’s α coefficient of Factor 15*	*0.60*
*Eigen values*	*1.401*
*Explained variance (%)*	*1.67*
*Cumulative variance (%)*	*60.61*
***Total test-re test of reinforcing factor structure***	***ICC = 0.964***
***Total test-re test of The MSSCQ***	***ICC = 0.94***
***Total Cronbach’s α coefficient of reinforcing factor structure***	***0.84***
***Cronbach’s alpha coefficient of The MSSCQ***	***0.92***

*Test re-test stability with a 2-week interval (n = 60).

### Confirmatory factor analysis

**Figure 2:**
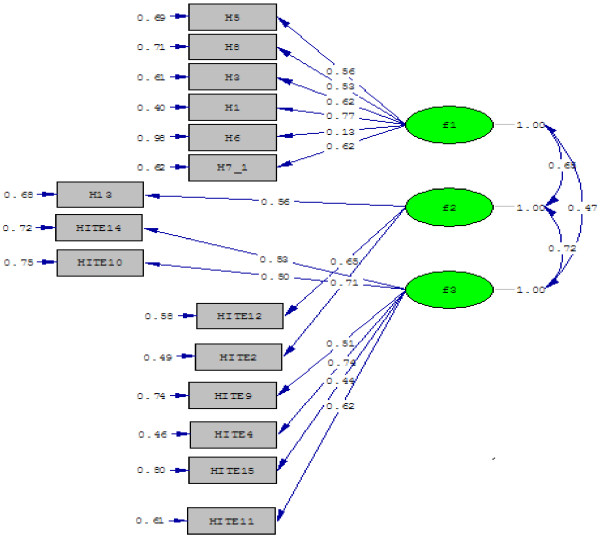
The results obtained from confirmatory factor analysis.

The 82-item questionnaire was subjected to the confirmatory factor analysis to determine a model with appropriate fitness. The pattern was revised for several times and an optimal pattern was eventually fitted and confirmed, as presented in Figure 2. The relative chi-square *(X*^*2*^*/df)* was equal to 2.64. It is indicated the fitness of the model. The RMSEA of the model was equal to 0.07 (90% CI = 0.066-0.089) indicating a good fit. The GFI, CFI, NNFI, NFI were more than 0.8 (0.9, 0.86, 0.83, 0.80 respectively) all of which fall in the acceptable range. The SRMR was less than 0.08 (0.06), indicating adequate fit and acceptable value. The standardized coefficient of the predisposing (f1 = factors 1, 3, 5- 8), the enabling (f2 = factors 2, 12-13) and the reinforcing factors (f3 = factors 4, 9, 11, 14-15) were compiled in Tables 2, 3, and 4 and also is shown in Figure 2.

### Reliability

The internal consistency of the MSSCQ as assessed by the Cronbach’s α coefficient showed satisfactory results. The value for alpha ranged from 0.84 to 0.89. The Intraclass Correlation Coefficient (ICC) of the MSSCQ also was found satisfactory, indicating that the questionnaire had a good stability. The results are shown in Tables 2, 3 and 4.

## Discussion

This study was reported the stages of designing and developing an instrument for assessing the factors associated with mother-newborn skin-to-skin immediately after birth based on the Precede-Proceed model. The results indicated satisfactory psychometric properties for the instrument with 3 constructs and 15 sub-scales and 82 statements. This is the first study that provides a measure for assessing the factors associated with skin-to-skin immediately after birth.

The statements for this instrument were prepared through a qualitative study with midwives working in labor rooms. In fact we first developed a 5-construct model derived from the qualitative section of the study, and then it was compared to the model derived from factor analysis, the results of which were not satisfactory. Therefore, we used the Precede-Proceed model to examine the fitness of the data. Finally, a 3-construct model with 15 factors and 82 statements was achieved. Similarly, Chiang et al. used the Precede-Proceed model in their study in 2003 [43].

The findings indicated that three factors from three different constructs had the highest predictive power in explaining skin-to-skin contact as derived from the confirmatory factor analysis. These were: midwife’s attitude (0.77) from predisposing construct, midwife’s encouragement from reinforcing construct (0.74), and preparations from enabling construct (0.71) (see Figure 2). In the following sections we will try to discuss about each construct separately.

### Predisposing construct

Six factors composed the predisposing construct. It is well known that predisposing construct precede behavior modification and motivate the person to perform the behavior and includes different factors including knowledge, beliefs, values, attitudes, personal beliefs and priorities, skills and self-confidence [19,43,44]. According to the pattern derived from the current study, newborn’s health, mother’s physical health and mental health were compatible with sub construct of attitude and all other factors were compatible with other sub constructs of the predisposing factors in the Precede-Proceed model. The greater role of ‘midwife’s attitude’ in predisposing construct in terms of predictive power may reflect the lack of skin contact in educational curriculum of midwives, leading to their poor knowledge of the subject and necessitating well-organized educational courses.

### Reinforcing construct

Reinforcing factors are factors that may facilitate continuation, repetition and stabilizing a given behavior. These include factors such as social support, peer group, family, authoritative individuals, employers, teachers, healthcare personnel, leaders, decision-makers and substitutes respected by the individual [19,43,44]. In our study, the factors of ‘medical team’s support’ and ‘support of mother’s companion’ were compatible with the sub constructs of social support and family, and the factor ‘midwife’s encouragement’ was compatible with the sub construct of healthcare personnel. Nevertheless, it appears that the statements of ‘midwife’s occupational satisfaction’ and ‘self-motivation’ were especially important due to the particular occupational situations of midwives in the Iranian society. However, with regard to the high predictive power of ‘midwife’s encouragement’ in the reinforcing construct, it might be argued that the numerous and irrelevant responsibilities imposed on midwives has caused their lack of interest in performing skin-to-skin contact.

### Enabling construct

Enabling factors pave the way to behavioral or environmental modifications that affect the person’s behavior directly or indirectly via environmental factors, such as regulations, laws, health plan, availability of services, access to necessary resources, and having the skills [19,43,44]. In this study, the managerial-planning factor was compatible with the sub construct of regulations and the factor ‘services provided for mother’ was compatible with the sub construct of availability of services. In our study, the second factor ‘preparation’ was not compatible with any of the sub constructs of enabling factors, which may be due to the specific cultural characteristics of the Iranian society.

The factor ‘preparations’ had a high predictive power in enabling construct. This may reflect the unawareness and indifference of other healthcare personnel including physicians and other staff, as well as the mother and her family, necessitating educational programs via different media including audiovisual training on radio, television, journals, booklets, pamphlets and other means. It appears that when the mother is highly aware of the importance of skin-to-skin and requires it from the healthcare team, the personnel will have greater motivation to perform it.

In the present study, performing both exploratory and factor analyses, the results indicated a good structure for the MSSCQ. Exploratory factor analysis indicated that the structure of the questionnaire jointly accounted for 60.61% of the total variance observed and the confirmatory factor analysis showed that the factor structure of the questionnaire was appropriate.

### Limitations

Although the study reported here benefits from several strengths, some limitations of the current project should be acknowledged. For instance, during the qualitative phase we felt that midwives are experiencing some difficulties in responding to our questions since senior staffs also were present. In addition, one should note that we used the same sample for exploratory and confirmatory factor analyses. This might limit the findings. Finally, we included 3 items (item 5, 6, and 10) in the questionnaire while in EFA the loading was less than 0.3. These were included due to the fact that midwives were very keen to include these items in the questionnaire.

## Conclusion

The Mother-Newborn Skin-to-Skin Contact Questionnaire (MSSCQ) is a reliable and valid theory-based measurement and now can be used in clinical practice, midwifery and nursing studies.

## Competing interests

The authors declare that they no competing interests.

## Authors’ contributions

FN was the main investigator, collected the data, performed the statistical analysis, and drafted the manuscript. SST provided assistance in the design of the study and participated in manuscript preparation. MH provided assistance in design and analysis. EH helped in statistical analysis. AM contributed to analysis, helped as a consultant and provided the final article. All authors read and approved the final manuscript.

## Pre-publication history

The pre-publication history for this paper can be accessed here:

http://www.biomedcentral.com/1471-2393/14/85/prepub

## Supplementary Material

Additional file 1 The file contains The Mother-Newborn Skin-to-Skin Contact Questionnaire (MSSCQ). Click here for file
