# Supplementary material for: The Mother-Newborn Skin-to-Skin Contact Questionnaire (MSSCQ): development and psychometric evaluation among Iranian midwives
Source: BMC Pregnancy Childbirth. 2014 Feb 24;14:85. doi: 10.1186/1471-2393-14-85 (PMC3937427; doi:10.1186/1471-2393-14-85)
Supplement: Additional file 1 — The file contains The Mother-Newborn Skin-to-Skin Contact Questionnaire (MSSCQ). [file 1471-2393-14-85-S1.doc]

The Mother-Newborn Skin-to-Skin Contact Questionnaire (MSSCQ)

| **Predisposing factors** | | ***Agree***  ***(2)*** | | ***Neither agree nor disagree***  ***(1)*** | | | | ***Disagree***  ***(0)*** | |  |
| --- | --- | --- | --- | --- | --- | --- | --- | --- | --- | --- |
| **Midwife’s attitude** | |  | |  | | | |  | |  |
| 1. Skin contact improves mother’s physical health. | |  | |  | | | |  | |  |
| 2. Skin contact improves neonate’s physical health. | |  | |  | | | |  | |  |
| 3. Skin contact makes mother take better care of the child. | |  | |  | | | |  | |  |
| 4. Skin contact improves mother’s success in breastfeeding. | |  | |  | | | |  | |  |
| 5. Skin contact improves mother’s satisfaction. | |  | |  | | | |  | |  |
| 6. Skin contact improves mother’s mental health. | |  | |  | | | |  | |  |
| 7. Skin contact establishes verbal/emotional bonding between midwife and mother. | |  | |  | | | |  | |  |
| 8. Skin contact creates a sense of security in the newborn. | |  | |  | | | |  | |  |
| 9. Skin contact enhances mother’s love for the newborn. | |  | |  | | | |  | |  |
| 10. Skin contact reduces mother’s stress. | |  | |  | | | |  | |  |
| 11. Being skilled in performing skin contact by midwife improves the results. | |  | |  | | | |  | |  |
| **Newborn’s health** | |  | |  | | | |  | |  |
| 12. Skin contact improves newborn’s immunity system. | |  | |  | | | |  | |  |
| 13. Skin contact improves the development of the newborn. | |  | |  | | | |  | |  |
| 14. Skin contact establishes an emotional bond between parents and the newborn. | |  | |  | | | |  | |  |
| 15. Skin contact regulates the newborn’s blood oxygen level. | |  | |  | | | |  | |  |
| 16. Skin contact regulates the newborn’s heartbeat. | |  | |  | | | |  | |  |
| 17. Skin contact improves the newborn’s breathing. | |  | |  | | | |  | |  |
| 18. Skin contact regulates the newborn’s body temperature. | |  | |  | | | |  | |  |
| **Mother’s physical health** | |  | |  | | | |  | |  |
| 19. Skin contact accelerates placental delivery. | |  | |  | | | |  | |  |
| 20. Skin contact accelerates the uterus’s return to normal. | |  | |  | | | |  | |  |
| 21. Skin contact promotes oxytocin release in mother. | |  | |  | | | |  | |  |
| 22. Skin contact reduces post-labor bleeding. | |  | |  | | | |  | |  |
| **Midwife’s belief about obstacles of performing skin contact** | |  | |  | | | |  | |  |
| 23. The newborn’s ill situation hinders skin contact. | |  | |  | | | |  | |  |
| 24. Skin contact is not feasible for ill mothers. | |  | |  | | | |  | |  |
| 25. Problems of mothers undergoing C-section affect skin contact. | |  | |  | | | |  | |  |
| 26. Problems of neonates born to C-section affect skin contact. | |  | |  | | | |  | |  |
| 27. Mother’s fatigue caused by nonstandard intervention during labor affects skin contact. | |  | |  | | | |  | |  |
| **Midwife’s belief in self-efficacy** | |  | |  | | | |  | |  |
| 28. I believe skin contact is essential. | |  | |  | | | |  | |  |
| 29. I believe skin contact entails positive results. | |  | |  | | | |  | |  |
| 30. I believe skin contact is important. | |  | |  | | | |  | |  |
| 31. I believe I can perform skin contact with minimum facilities. | |  | |  | | | |  | |  |
| 32. I believe my recommendations for skin contact are acceptable for the mother. | |  | |  | | | |  | |  |
| 33. I believe I can use my knowledge to perform skin contact. | |  | |  | | | |  | |  |
| 34. I believe in positive results of the skin contact and I perform it. | |  | |  | | | |  | |  |
| **Mental health** | |  | |  | | | |  | |  |
| 35. Skin contact establishes an emotional bond between mother and newborn. | |  | |  | | | |  | |  |
| 36. Skin contact improves the acceptance of motherhood role by the mother. | |  | |  | | | |  | |  |
| 37. Skin contact creates a sense of security in mother and newborn. | |  | |  | | | |  | |  |
| 38. Skin contact results in future attachment between mother and child. | |  | |  | | | |  | |  |
| Enabling factors | | ***Agree***  ***(2)*** | | | ***Neither agree nor disagree***  ***(1)*** | | ***Disagree***  ***(0)*** | | |  |
| **Managerial-planning** | |  | | |  | |  | | |  |
| 1. Presence of a supportive program in the ministry improves skin-to-skin contact. | |  | | |  | |  | | |  |
| 2. Skill-teaching programs in hospital improve skin-to-skin contact. | |  | | |  | |  | | |  |
| 3. Placing skin-to-skin contact in policies of the ministry of health will improve its implementation. | |  | | |  | |  | | |  |
| 4. Encouraging the midwife by hospital authorities will improve skin-to-skin contact. | |  | | |  | |  | | |  |
| **Service provided to mother** | |  | | |  | |  | | |  |
| 5. Physiologic delivery has a positive impact on skin-to-skin contact. | |  | | |  | |  | | |  |
| 6. Encouraging the mother to have skin contact in labor room will improve skin-to-skin contact. | |  | | |  | |  | | |  |
| 7. Collaboration of the labor-supporting team improves skin-to-skin contact. | |  | | |  | |  | | |  |
| 8. Availability of adequate human resources in labor room improves skin-to-skin contact. | |  | | |  | |  | | |  |
| 9. Professional ethical commitment of the midwife improves skin-to-skin contact. | |  | | |  | |  | | |  |
| **Preparations** | |  | | |  | |  | | |  |
| 10. Educating mothers during pregnancy improves skin-to-skin contact. | |  | | |  | |  | | |  |
| 11. Educating companions improves skin-to-skin contact. | |  | | |  | |  | | |  |
| 12. Educating the parents before pregnancy improves skin-to-skin contact. | |  | | |  | |  | | |  |
| 13. Legalizing skin-to-skin contact improves its implementation in hospitals. | |  | | |  | |  | | |  |
| 14. Including skin-to-skin contact in educational curricula of medical and midwifery students will improve its implementation. | |  | | |  | |  | | |  |
| 15. Mandating skin-to-skin contact to all hospitals will improve its implementation. | |  | | |  | |  | | |  |
| 16. Placing a point for skin-to-skin contact in ranking of hospitals will improve its implementation. | |  | | |  | |  | | |  |
| 17. Developing regulations for evaluating midwives based on skin-to-skin contact will improve its implementation. | |  | | |  | |  | | |  |
| 18. The supervision of authorities on correct skin-to-skin contact will improve its implementation. | |  | | |  | |  | | |  |
|  | Reinforcing factors | | ***Agree***  ***(2)*** | | | ***Neither agree nor disagree***  ***(1)*** | | | ***Disagree***  ***(0)*** | |
|  | **Encouraging factors for midwives** | |  | | |  | | |  | |
|  | 1. Encouraging colleagues improves skin-to-skin contact. | |  | | |  | | |  | |
|  | 2. Patient’s confidence in the delivery team improves skin-to-skin contact. | |  | | |  | | |  | |
|  | 3. Mother’s calmness during skin-to-skin contact will encourage the midwife. | |  | | |  | | |  | |
|  | 4. Newborn’s calmness during skin-to-skin contact will encourage the midwife. | |  | | |  | | |  | |
|  | 5. Mother’s satisfaction with skin-to-skin contact will encourage the midwife. | |  | | |  | | |  | |
|  | 6. Mother’s desire for skin-to-skin contact will encourage the midwife. | |  | | |  | | |  | |
|  | 7. Mother’s request for skin-to-skin contact will encourage the midwife to perform it. | |  | | |  | | |  | |
|  | **Support of the medical team** | |  | | |  | | |  | |
|  | 8. Physician’s support will improve skin-to-skin contact. | |  | | |  | | |  | |
|  | 9. Anesthesiologist’s support will improve skin-to-skin contact. | |  | | |  | | |  | |
|  | 10. Pediatrician’s support will improve skin-to-skin contact. | |  | | |  | | |  | |
|  | 11. Hospital authorities’ support will improve skin-to-skin contact. | |  | | |  | | |  | |
|  | **Companion’s support** | |  | | |  | | |  | |
|  | 12. Presence of educated companion in the labor room improves skin-to-skin contact. | |  | | |  | | |  | |
|  | 13. Support of mother’s relatives improves skin-to-skin contact. | |  | | |  | | |  | |
|  | 14. The husband’s support improves skin-to-skin contact. | |  | | |  | | |  | |
|  | **Self-motivation** | |  | | |  | | |  | |
|  | 15. Midwife’s awareness of advantages of skin-to-skin contact improves its implementation. | |  | | |  | | |  | |
|  | 16. Midwife’s desire for skin-to-skin contact will encourage her to perform it. | |  | | |  | | |  | |
|  | 17. Awareness of advantages of skin-to-skin contact through media will improve its implementation. | |  | | |  | | |  | |
|  | 18. Midwife’s support for skin contact will encourage its implementation. | |  | | |  | | |  | |
|  | **Facilities and equipment** | |  | | |  | | |  | |
|  | 19. Presence of an appropriate labor bed affects skin contact. | |  | | |  | | |  | |
|  | 20. The temperature of the labor room affects skin contact. | |  | | |  | | |  | |
|  | 21. Availability of private space during labor affects skin contact. | |  | | |  | | |  | |
|  | 22. Presence of an appropriate space in the operation room affects skin contact. | |  | | |  | | |  | |
|  | 23. Presence of a midwife to take care of the newborn affects skin contact. | |  | | |  | | |  | |
|  | **Midwife’s occupational satisfaction** | |  | | |  | | |  | |
|  | 24. Midwife’s occupational satisfaction affects skin contact. | |  | | |  | | |  | |
|  | 25. Eliminating the marginal responsibilities of midwives affects skin contact. | |  | | |  | | |  | |
|  | 26. Providing independence and granting the responsibility of normal delivery to midwife affects skin contact. | |  | | |  | | |  | |

© Fatemeh Nahidi, 2014
